# Retraction Note: Exosomal DLX6-AS1 from hepatocellular carcinoma cells induces M2 macrophage polarization to promote migration and invasion in hepatocellular carcinoma through microRNA-15a-5p/CXCL17 axis

**DOI:** 10.1186/s13046-022-02353-x

**Published:** 2022-04-09

**Authors:** Lin-pei Wang, Jing Lin, Xiao-qiu Ma, Dong-yao Xu, Chun-feng Shi, Wei Wang, Xiao-jie Jiang

**Affiliations:** 1 Department of Hepatobiliary and Pancreatic Surgery, The Second Affiliated Hospital of Fujian Medical University, Quanzhou, 362000 Fujian China; 2 Department of Pathology, Affiliated Putian Hospital of Putian College, Putian, 363100 Fujian China; 3 Department of Internal Medical Oncology, The 910th Hospital of the People’s Liberation Army, Quanzhou, 362000 Fujian China; 4 Department of Hepatobiliary Surgery, Affiliated Putian Hospital of Putian College, Putian, 363100 Fujian China

**Retraction Note: J Exp Clin Cancer Res 40, 177 (2021)**

**https://doi.org/10.1186/s13046-021-01973-z**

The Editor in Chief has retracted this article. After concerns were raised regarding the western blots in the article the Authors were asked to provide the images of the original blots, however, the authors were unable to provide the whole film for each blot. The Editor-in-Chief therefore no longer has confidence in the presented results and the conclusions drawn in this study.

Lin-pei Wang, Jing Lin, Xiao-qiu Ma, Chun-feng Shi, Wei Wang & Xiao-jie Jiang do not agree to this retraction. Dong-yao Xu has not responded to any correspondence from the editor/publisher about this retraction.

